# Entering a new era for exercise countermeasures in human spaceflight

**DOI:** 10.1113/EP093248

**Published:** 2025-10-19

**Authors:** Enrico De Martino, Patrick Swain, Kirsty Lindsay, Claire Bruce‐Martin, Ewoud Jacobs, Bradley Barbour, Chris Buckley, Adam C. McDonnell, Nick Caplan, Luke Hughes

**Affiliations:** ^1^ Center for Neuroplasticity and Pain (CNAP), Department of Health Science and Technology, Faculty of Medicine Aalborg University Aalborg Denmark; ^2^ Aerospace Medicine and Rehabilitation Laboratory, School of Sport, Exercise and Rehabilitation, Faculty of Health and Wellbeing Northumbria University Newcastle upon Tyne UK

**Keywords:** exercise countermeasure, human spaceflight, lumbar spine, neuromuscular training, sensorimotor control

1

Since the beginning of human spaceflight, in‐flight exercise has been a cornerstone of space exploration to preserve crew health and facilitate mission success (Scott et al., 2019). Future long‐duration space exploration missions beyond low Earth orbit, such as to the Moon, will require exercise countermeasures that extend beyond the present systems available on the International Space Station (ISS) (Fernandez‐Gonzalo et al., 2025). Devices such as the T2 treadmill, CEVIS (cycle ergometer with vibration isolation and stabilization system) and ARED (the advanced resistive exercise device) (Figure 1a–c) have proved to be somewhat effective in preserving muscle mass and bone health in microgravity (Petersen et al., 2016). Unlike on the ISS, however, crewed missions beyond low Earth orbit will require crews to self‐manage exercise in extremely confined environments, requiring countermeasures that minimize set‐up time, operate reliably with minimal maintenance, support autonomous use, integrate multiple exercise modalities within a single device and require minimal energy consumption (Scott et al., 2023). Even on the ISS, exercise hardware has experienced periods of downtime and malfunction, underscoring the need for durable, rugged systems that are simple to operate. Within this context, the ‘novel exercise hardware for exploration’ (NEX4EX) device emerges as a particularly promising solution (Boecker et al., 2025). In addition to resistive and plyometric training, the NEX4EX device engages neuromuscular control responses within a single compact device, a feature especially relevant for mitigating neuromuscular deconditioning during crewed missions, while reducing mass, power and crew time, a combination not currently combined in ISS hardware (Figure 1d).

**FIGURE 1:**
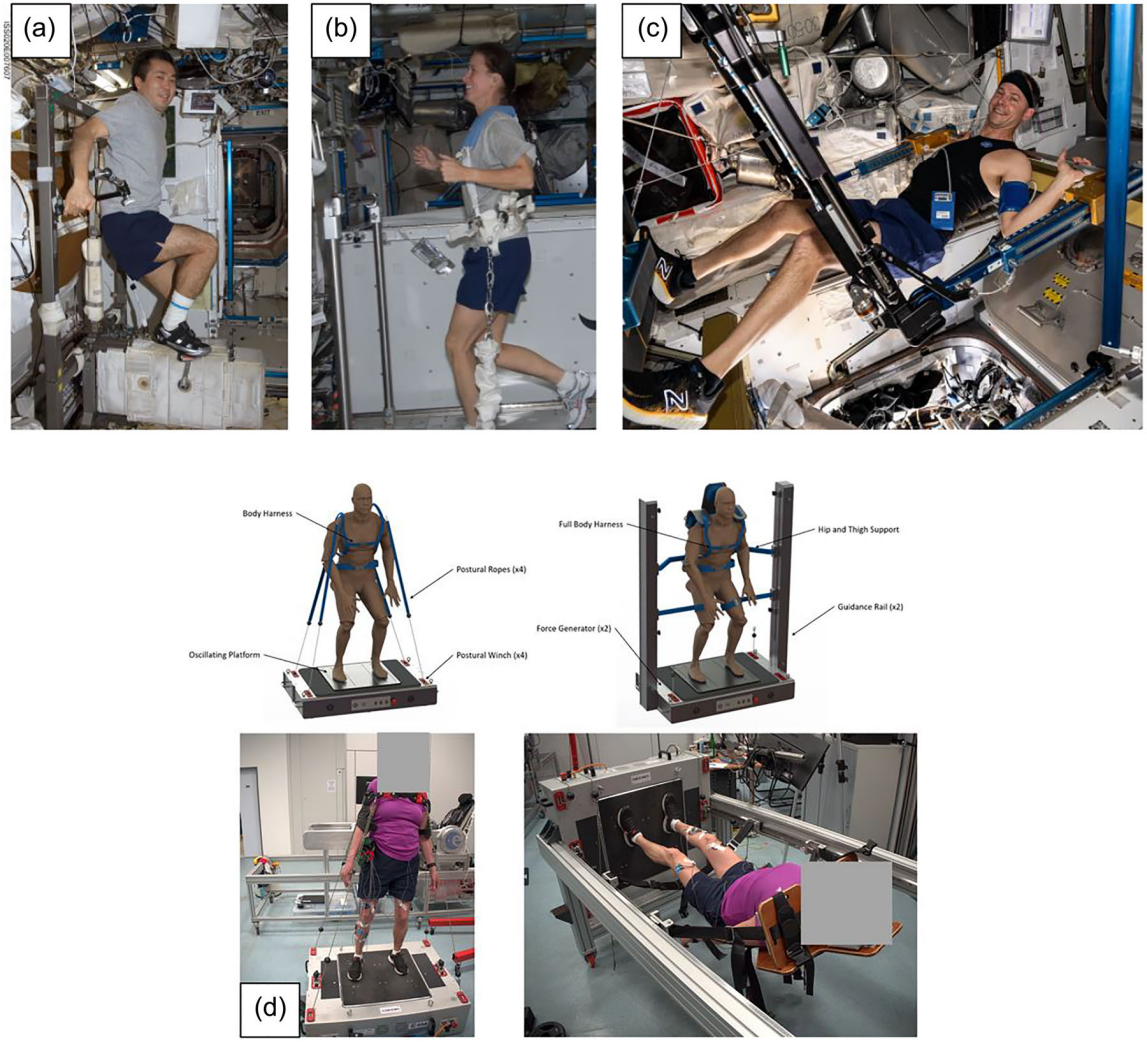
Overview of current exercise countermeasures on the International Space Station, including CEVIS (a), T2 treadmill (b), ARED (c) and the NEX4EX device (d). Abbreviations: ARED, advanced resistive exercise device; CEVIS, cycle ergometer with vibration isolation and stabilization system; NEX4EX, novel exercise hardware for exploration. Images (a–c) obtained from the NASA Image and Video Library; image (d) obtained with permission from Böcker et al. (2025) at https://creativecommons.org/licenses/by/4.0/.

The human body has evolved to counteract the constant force of gravity on Earth, developing physiological strategies that enable upright posture and locomotion (Niemitz, 2010); for example, the vertebral column and the muscles facilitate an upright posture by resisting axial (1 Gz) gravitational acceleration, which has been evolutionarily advantageous (Richardson, 2002). However, the antigravity structures are especially vulnerable to deconditioning during exposure to microgravity (Richter et al., 2017) and existing countermeasures primarily target large muscle groups, such as the quadriceps, gastrocnemius and gluteal muscles, providing only limited stimulation of the deep lumbar muscles, which are essential for stability and spinal health in the upright posture and during locomotion (Green & Scott, 2018). Currently, no dedicated exercise device is capable of directly training the deep paraspinal muscles, and specific reconditioning of these muscles is needed upon return to Earth (Lambrecht et al., 2017). The NEX4EX device potentially addresses this gap through its ability to induce controlled perturbations, thereby engaging motor responses that are fundamental for neuromuscular control.

The lumbar muscles, including the deep fibres of the lumbar multifidus, are crucial for spinal stability (Hodges et al., 2013). The lower lumbar spine is particularly vulnerable to microgravity‐induced deconditioning. Imaging and electrophysiological studies demonstrate atrophy/degeneration of the multifidus at the L4–L5 and L5–S1 intervertebral levels during unloading (De Martino et al., 2022), in addition to muscle delay during anticipatory postural adjustments (De Martino et al., 2021). This vulnerability might explain why astronauts frequently experience low back pain in‐flight, with reports indicating an incidence of ≤70%. The pain is typically dull and localized to the lower lumbar region and is sometimes severe enough to interfere with their daily activities (Pool‐Goudzwaard et al., 2015). Such findings mirror observations in terrestrial low back pain populations, where multifidus atrophy, fatty infiltration and impaired motor control are frequently reported (Hides et al., 2019; MacDonald et al., 2009). Importantly, terrestrial rehabilitation research has highlighted that targeted training of the deep trunk muscles, using progressive loading combined with controlled perturbations, can restore neuromuscular control and significantly reduce recurrence of pain and injury episodes (Hodges et al., 2013).

Based on this evidence, the European Space Agency applies a similar approach during postflight reconditioning, when physiotherapists emphasize motor control retraining, progressive spinal loading and perturbation‐based exercises to restore spinal function and neuromuscular control (Lambrecht et al., 2017). Perturbation‐based approaches to stimulate the lumbar spine might be particularly important in microgravity, because reduced gravity decreases stabilizing responses of deep lumbar paraspinal muscles (De Martino et al., 2020). In this context, the ability of NEX4EX to deliver controlled perturbations during exercise is highly relevant. By eliciting reflexive stabilizing responses, the device might help to maintain activation of the deep spinal muscles in microgravity. This dual focus on strength and neuromuscular control not only fills a gap in the present exercise countermeasure programme, but also aligns with principles already validated in terrestrial rehabilitation.

However, several aspects require further work. For instance, translating upright reference forces to horizontal and in‐flight configurations will require explicit load mapping, with verification in analogues (e.g., bed rest, centrifuge, whole‐body suspension) and in parabolic flight. In addition, because existing reports provide limited guidance on dose (repetitions, sets and weekly volume), defining an exploration‐ready prescription should be a priority. Furthermore, demonstrating that NEX4EX preserves conditioning of the lower‐limb multi‐joint muscle groups would strengthen the case for adoption. Early testing in three participants shows variability in percentage activation, contraction speed and range of motion compared with reference values, underscoring the need for comparative studies with larger, standardized datasets.

By combining resistive, plyometric and neuromuscular control exercises within a single device, NEX4EX represents a new era for exercise countermeasure technology, with the potential to mitigate critical risks of deconditioning during crewed missions beyond low Earth orbit. Further refinement and validation will help to demonstrate its role as an essential element of in‐flight exercise programmes, thereby safeguarding astronaut health and performance.

## AUTHOR CONTRIBUTIONS

Enrico De Martino and Luke Hughes conceptualized and wrote the manuscript. Patrick Swain, Kirsty Lindsay, Claire Bruce‐Martin, Ewoud Jacobs, Bradley Barbour, Chris Buckley, Adam C. McDonnell, and Nick Caplan reviewed and approved the final draft. All authors approved the final version of the manuscript and agree to be accountable for all aspects of the work in ensuring that questions related to the accuracy or integrity of any part of the work are appropriately investigated and resolved. All persons designated as authors qualify for authorship, and all those who qualify for authorship are listed.

## CONFLICT OF INTEREST

None declared.

## FUNDING INFORMATION

None.
